# The Relationship between Weight Stigma, Physical Appearance Concerns, and Enjoyment and Tendency to Avoid Physical Activity and Sport

**DOI:** 10.3390/ijerph18199957

**Published:** 2021-09-22

**Authors:** Nadia Bevan, Kerry S. O’Brien, Chung-Ying Lin, Janet D. Latner, Brian Vandenberg, Ruth Jeanes, Rebecca M. Puhl, I-Hua Chen, Simon Moss, Georgia Rush

**Affiliations:** 1 School of Social Sciences, Faculty of Arts and Faculty of Education, Monash University, Melbourne, VIC 3800, Australia; nadia.bevan@monash.edu (N.B.); brian.vandenberg@monash.edu (B.V.); ruth.jeanes@monash.edu (R.J.); georgia.rush@monash.edu (G.R.); 2 Institute of Allied Health Sciences, College of Medicine, National Cheng Kung University, Tainan 701401, Taiwan; cylin36933@gmail.com; 3 Department of Public Health, National Cheng Kung University Hospital, College of Medicine, National Cheng Kung University, Tainan 701401, Taiwan; 4 Department of Occupational Therapy, College of Medicine, National Cheng Kung University, Tainan 701401, Taiwan; 5 Department of Psychology, University of Hawaii, Honolulu, HI 96822, USA; jlatner@hawaii.edu; 6 Department of Human Development and Family Sciences, University of Connecticut, Storrs, CT 06269, USA; rebecca.puhl@uconn.edu; 7 Chinese Academy of Education Big Data, Qufu Normal University, Qufu 273165, China; ahole.chen@gmail.com; 8 College of Health and Human Sciences, Charles Darwin University, Casuarina 0815, Australia; simon.moss@cdu.edu.au

**Keywords:** weight stigma, appearance evaluation, physical activity participation, sport participation, physical activity enjoyment, sport, physical activity avoidance, obesity

## Abstract

Participation in physical activity and sport is on the decline and there is a poor understanding of the psychosocial factors that contribute to people’s reluctance to participate. We examined whether there were relationships between factors such as weight stigma, weight bias internalization, appearance evaluation, and fears of negative appearance evaluations, and enjoyment and avoidance of physical activity and sport. Undergraduate students (*N* = 579) completed a survey assessing demographics, and the variables described above. In hierarchal multivariate regression models, weight stigma (*β* = −0.16, *p* < 0.001), appearance evaluation (*β* = 0.19, *p* = 0.001), and weight bias internalization (*β* = −0.19, *p* = 0.003) were associated with lower enjoyment of physical activity and sport. Weight stigma (*β* = 0.46, *p* = 0.001), weight bias internalization (*β* = 0.42, *p* = 0.001), and fear of negative appearance evaluations (*β* = 0.16, *p* = 0.000) were also significantly associated with the tendency to avoid physical activity and sport. Serial mediation analysis showed the relationship between weight stigma and enjoyment of physical activity and sport was through appearance evaluation and weight bias internalization (indirect effect = −0.007, *SE* = 0.002, 95% CI = −0.01, −0.02). Similarly, the relationship between weight stigma and avoidance of physical activity and sport was through weight bias internalization and fear of negative appearance evaluations (indirect effect = 0.11, *SE* = 0.03, 95% CI = 0.05, 0.16). These results suggest that weight stigma and concerns about one’s physical appearance influence people’s enjoyment and reasoning for avoiding physical activity and sport. Research is needed to identify ways to reduce body-related stigma and increase enjoyment and participation in physical activity and sport.

## 1. Introduction

Regular participation in physical activity and sport is important for the prevention of numerous mental and physical health conditions [1,2]. For example, people who regularly participate in formal exercise or sport are less likely to develop type 2 diabetes, coronary heart disease, obesity, major depression and anxiety disorders, and some cancers [3]. Accordingly, public health experts and governments have been trying to increase regular participation in physical activity and sport for over three decades. Despite increased research, public health efforts, and investment by governments, participation in physical activity and sport continues to decline in most Western nations, and solutions for addressing this problem have not been identified [4,5,6,7].

Research on the role of various environmental and socio-economic factors suggests that high cost, poor accessibility, and lack of leisure time are barriers to physical activity and sport participation [8,9]. However, little research has examined the relationship between psychosocial influences such as weight-related stigma, physical appearance-related concerns (body image, fear of negative appearance evaluations) and enjoyment and avoidance of physical activity and sport [10,11]. Previous research suggests that higher rates of perceived weight stigma (e.g., teasing, negative verbal commentary) may be related to exercise avoidance in people with higher body weight [12,13]. For example, Vartanian and colleagues [14,15] found that experiences of weight stigma were associated with decreased motivation to exercise. This relationship may be particularly evident in women who have significantly lower levels of physical activity, sport participation, and exercise intentions, and higher rates of body dissatisfaction [6,16]. Weight stigma has also been found to be associated with lowered physical activity intention among young adults [10,17]. Similarly, research by Jackson and Steptoe [18] found that weight-based discrimination was reported as a barrier to physical activity, independent of body mass index (BMI) [19].

Experiences of weight stigma (e.g., teasing, negative verbal comments) and internalized weight stigma (e.g., self-stigma and blame, weight bias internalization, disgust) have been found to be associated with negative psychological outcomes, including greater body dissatisfaction, anxiety, depression [20,21,22,23,24], and poorer physical health [25]. Internalized weight stigma in particular has been found to be consistently associated with poorer health outcomes (e.g., weight gain, poorer psychosocial well-being) [26]. Related research also suggests that appearance-related concerns (e.g., body image) have a role in exercise avoidance [11], with appearance-related concerns associated with less motivation to exercise [27]. However, little research has examined the relationships between weight stigma, appearance related concerns, and enjoyment or avoidance of physical activity and sport. Specifically, it is unknown whether the relationship between experiences of weight stigma and enjoyment and avoidance of physical activity and sport are mediated by factors such as weight bias internalization, appearance-related concerns, and/or fear of negative appearance evaluations.

Although there is no research directly examining these interrelationships, previous research suggests that such relationships may exist. For example, a cross-sectional study found that experiences of weight stigma and weight stigma internalization were related to less exercise and lower exercise self-efficacy, respectively, and the relationship between weight stigma experiences and exercise was mediated by weight stigma internalization [12]. Vartanian and colleagues [28] similarly found significant relationships between body image concerns and motivation to exercise. Research examining fear of negative appearance evaluation and body image avoidance also suggests that concerns about one’s physical appearance and internalized weight stigma [12] may be related to avoidance of settings involving appraisals of one’s physical appearance (e.g., physical activity and sport). Research on the enjoyment of sport also suggests that there is a relationship between affect regarding sport and continued participation in sport [29].

This exploratory study examines potential relationships between weight stigma, weight stigma internalization, appearance-related concerns, and tendency to avoid participation in physical activity and sport and enjoyment of physical activity and sport. We also sought to examine whether relationships between weight stigma and tendency to avoid participation in physical activity and sport and enjoyment is mediated by appearance-related concerns.

## 2. Materials and Methods

### 2.1. Participants

Undergraduate students (*N* = 647) were invited to participate in the study in return for course credit. The majority (89.7%; *N* = 581) agreed to participate and provided responses to the survey. Two students provided no response to a question assessing their sex, leaving *N* = 579 students for inclusion in analyses. Post-hoc power calculations suggested the sample size was sufficient to provide >90% power to detect a small effect (0.2) at the 0.05 level of significance. Mean age of the sample was 19.8 years, with a standard deviation of 2.3 years. The majority of the sample were women (*N* = 428; 73.8%). Participant BMI (kg/m^2^) was calculated from self-reported weight and height (M = 22.5 kg/m^2^, SD = 3.44), with BMI’s ranging from 14.9 to 38.7. Approximately 10.2% of respondents were categorized as “underweight” by BMI (BMI < 18.5), 71.5% as “normal range” (BMI = 18.52–4.9), 14.3% as “overweight” (BMI = 25–29.9), and 3.5% as “obese” (BMI ≥ 30) [30]. Three participants were excluded from analysis as they did not provide height and/or weight information. We asked participants to provide their ethnicity via an open-ended written response. Approximately, 34% of participants specifically used the word white when defining their ethnicity. An additional 40% identified as having an Asian background, and 26% identified their country of origin (e.g., citizen status) rather than an ethnicity per se.

We collected demographic characteristics (age, sex, and weight and height) and administered measures of weight stigma, weight stigma internalization, appearance evaluation, fear of negative appearance evaluation, enjoyment of physical activity and sport, and the tendency to avoid physical activity and sport. Ethical approval was obtained from the host university’s ethics panel (ID: 8912).

### 2.2. Weight Stigma

Consistent with previous research [22,31], we used the modified five-item Perception of Teasing Scale [32] to measure weight stigma. Scale items have two parts, the first assessing frequency of teasing about weight, the second assessing how much the teasing hurt. Participants first rate how often they were teased in the past 12 months about their weight using a 5-point scale, where 1 = “never” and 5 = “very often”, and then how upset the teasing made them feel (1 = “not upset” and 5 = “very upset”). Participants responding 1 (“never”) to the first part of the question assessing frequency, had the second half of the item assessing level of upset coded as a 0. The items from the original scale were modified slightly to be relevant to all weight categories. Instead of using the term “heavy”, the term “weight” was inserted in items so as to be applicable to participants of diverse weight categories. For example, the first item in the original scale asked participants to rate how often “People made fun of you because you were heavy”, whereas, in the modified scale this item stated, “People make fun of you because of your weight” [22]. The weight stigma frequency and upset items were highly correlated with one another (*r*s = 0.82 to 0.84), and an examination of factor structure showed a single factor (eigen value = 6.84). Accordingly, a weight stigma score was calculated by combining the total of the weight stigma-frequency and upset components and dividing by two. Cronbach’s alpha for the measure in this sample was 0.93. Weight stigma scores ranged from 2.5 to 25, with higher scores indicating greater weight stigmatization.

### 2.3. Weight Bias Internalisation

Weight stigma internalization was measured using the 11 item Weight Bias Internalization Scale (WBIS) [33], modified to use the term “weight” instead of “overweight” [34] to be applicable to participants of diverse body sizes. This scale assesses the degree to which people accept and internalize negative weight-related messages, stereotypes, and attitudes. Participants are asked to indicate the extent to which they agree with statements about themselves (e.g., “My weight is a major way that I judge my value as a person”) using a 7-point scale (1 = “strongly disagree” and 7 = “strongly agree”). A mean score of all 11 items is calculated for each participant, with WBIS scores ranging from 1 and 7, with higher scores indicating greater weight bias internalization. Cronbach’s alpha for the present study was 0.93.

### 2.4. Appearance Evaluation

We used the appearance evaluation scale of the Multidimensional Body-Self Relations Questionnaire [35] to assess participants’ feelings and perceptions of their own physical attractiveness (body image). The scale has 7 items, and participants indicate their agreement with statements such as, “My body is sexually appealing” using a scale ranging from 1 = strongly disagree to 5 = strongly agree. Cronbach’s alpha for the appearance evaluation scale was 0.85. Higher scores on this scale indicate greater satisfaction with one’s physical appearance.

### 2.5. Fear of Negative Appearance Evaluation

The Fear of Negative Appearance Evaluation Scale (FNAE) [36] is a 6-item scale assessing people’s fear or worry regarding the potential of others to make evaluations of their physical appearance. Participants were asked to answer what comes closest to how they feel (e.g., “It bothers me if I know someone is judging my physical shape”) using a 5-point scale (1 = “not at all” and 5 = “extremely”). Cronbach’s alpha for the present study was 0.94.

### 2.6. Enjoyment of Physical Activity and Sport

The Physical Activity Enjoyment Scale [37,38] was used to assess enjoyment of physical activity and sport. Originally, an 18-item measure, subsequent research identified a modified 16-item version showing a single factor measure of enjoyment of physical activity [38]. A limitation of the measure is that it assesses one specific activity (e.g., tennis) and at that current point in time, instead of assessing enjoyment of physical activity, exercise or sport across a number of activities. Accordingly, we modified the opening statement introducing the scale to: “The following scale asks about how you generally feel when you are engaged in physical exercise for fitness and health (e.g., running, playing sport, going to the gym). Please indicate how much you agree or disagree with each statement below.” Consistent with previous research [38], a 5-point Likert scale was used to indicate participants agreement and disagreement (1 = totally agree to 5 = totally disagree) with the 16 statements assessing level of enjoyment (e.g., I enjoy it; It frustrates me; I get something out of it). Higher mean scores indicate more enjoyment when engaged in physical exercise and sport. Confirmatory factor analysis showed the scale retained a factor solution for enjoyment (eigen value = 8.87). Similarly, scale internal consistency was good (α = 0.94).

### 2.7. Tendency to Avoid Physical Activity and Sport

Because there is no existing measure assessing the Tendency to Avoid Physical Activity and Sport (TAPAS) due to weight or appearance related concerns, we developed a 10-item scale to assess this construct [39]. This scale included items such as “I avoid participating in sport because of my fear of being judged about my physical appearance” and “I avoid physical activity because I don’t like how my body looks when exercising”. Participants indicated their agreement or disagreement using a five-point scale ranging from 1 = strongly disagree to 5 = strongly agree. Exploratory factor analysis revealed a single factor solution for the scale (eigen value = 7.43) with a Cronbach’s alpha of 0.95. The scores from ten items of the TAPAS are summed to provide a scale score ranging from 10–50, with higher scores indicating greater avoidance of physical activity and sport.

### 2.8. Analysis

Independent *t*-tests were used to examine mean differences between men and women on the variables of interest. We examine differences between men and women because of literature demonstrating consistent sex differences in body image, weight stigma, and participation in sport. Pearson’s correlation coefficients were used to examine simple bivariate relationships. Hierarchical multivariate linear regression models were used to test relationships between variables of interest and enjoyment of physical activity and sport, and avoidance of physical activity and sport, respectively. In the first model (step) of regression models, we entered age, sex, and BMI. Weight stigma experiences was entered in the second model, and appearance evaluation and weight stigma internalization in the third model. Fear of negative appearance evaluation was added in a final model 4. The PROCESS macro (Model 6) for SPSS (IBM Corp, Armonk, NY, USA) was used to run serial mediation analyses [40]. We report coefficients and mediation for all models. Statistical significance was set at the < 0.05 level. Note, exploratory moderated mediation analysis with sex and BMI as moderators and the pattern of results did not vary as a function of sex or BMI.

## 3. Results

As can be seen in Table 1, women reported significantly higher weight stigma, WBIS, and FNAE scores than men. Women also reported significantly lower appearance evaluation, enjoyment of physical activity and sport, and higher levels of tendency to avoid physical activity and sport.

### 3.1. Bivariate Analyses

Table 2 displays the correlation coefficients for the variables of interest. For both men and women, weight stigma, WBIS, appearance evaluation, FNAE, enjoyment of physical activity and sport, and tendency to avoid physical activity and sport were all significantly correlated with each other. Higher levels of weight stigma, weight bias internalization, and fear of negative appearance evaluation were associated with lower enjoyment of physical activity and sport, and greater tendency to avoid physical activity and sport. Lower appearance evaluation scores were associated with lower enjoyment and with higher tendency to avoid physical activity and sport.

### 3.2. Regression Models

The results of the hierarchical multivariate regression analyses are displayed in Table 3. All models explained significant amounts of variance in enjoyment of physical activity and sport, and tendency to avoid physical activity and sport scores (ps < 0.0001). For enjoyment of physical activity and sport, sex was the only significant predictor in the first model. In the second model, weight stigma explained a significant additional proportion of variance in enjoyment scores. The entry of appearance evaluation and WBIS in a third model explained an additional 8% of variance in enjoyment of physical activity and sport. However, weight stigma was no longer a significant predictor, suggesting that the relationship between weight stigma and enjoyment of physical activity and sport is explained by appearance evaluations and weight stigma internalization. Additionally, the relationship between sex and enjoyment of physical activity and sport was reduced in the presence of appearance evaluation and WBIS scores. In the final model, entry of FNAE did not explain any additional variance in enjoyment of physical activity and sport scores.

For the tendency to avoid physical activity and sport, age, sex, and BMI accounted for 10% of the variance in the first model, with sex and BMI as significant predictors of the tendency to avoid physical activity and sport scores. In the second model, the addition of weight stigma accounted for an additional 20% of the variance in avoidance scores. In the third model, appearance evaluation and WBIS accounted for another 14% of the variance in the tendency to avoid physical activity and sport scores. However, appearance evaluation was not a significant predictor. In the final model, entry of FNAE accounted for a small but significant additional proportion of variance in avoidance of physical activity and sport scores.

### 3.3. Mediation Analysis

**Figure 1:**
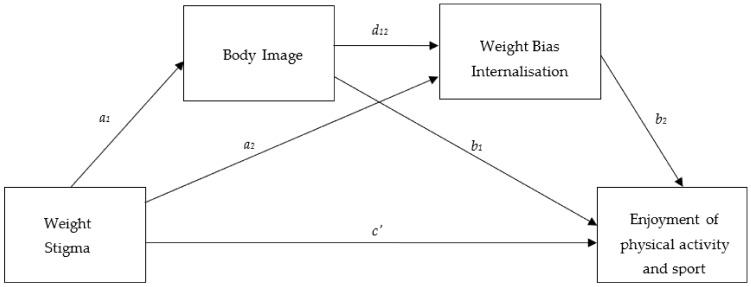
Conceptual Mediation Model. Indirect effect of weight stigma on enjoyment of physical activity and sport through body image only = *a*_1_, *b*_1_; indirect effect of weight stigma on enjoyment of physical activity and sport through body image and weight bias internalization in serial = *a*_1_, *d*_12_, *b*_2_; direct effect on weight stigma of enjoyment of physical activity and sport = *c*_1._

**Figure 2:**
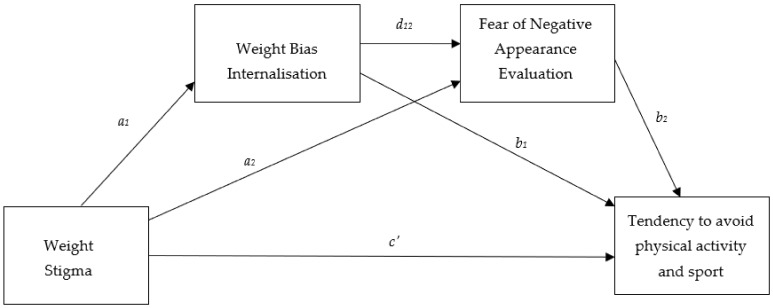
Conceptual Mediation Model. Indirect effect of weight stigma on tendency to avoid physical activity and sport through weight bias internalization only = *a*_1_, *b*_1_; indirect effect of weight stigma on tendency to avoid physical activity and sport through weight bias internalization and fear of negative appearance evaluation in serial = *a*_1_, *d*_12_, *b*_2_; direct effect of weight stigma on tendency to avoid physical activity and sport = *c*_1_.

Mediation models controlling for age, sex, and BMI examined the role of appearance evaluation, weight bias internalization, and fear of negative appearance evaluation in mediating the relationship between weight stigma and enjoyment and tendency to avoid physical activity and sport. The mediation models only included the mediation variables that were initially significant in the multivariate regression models (Table 3). Table 4 displays the results of the two serial mediation analyses. Both mediation models were statistically significant and accounted for differences in age, BMI, and sex (*p* < 0.001). The first model tested whether the relationship between weight stigma and enjoyment of physical activity and sport was through appearance evaluation and WBIS (Figure 1). All paths indicated in Figure 1 were significant, and there was a significant indirect path through appearance evaluation and WBIS (coefficient = −0.007, *SE* = 0.002, 95% CI = −0.01, −0.02) indicating serial mediation. That is, the relationship between weight stigma and enjoyment of physical activity and sport was through appearance evaluation and WBIS. The second mediation model (Figure 2) examined whether WBIS and FNAE mediated the relationship between weight stigma and the tendency to avoid physical activity and sport. Again, there was a significant indirect path through WBIS and FNAE (coefficient = 0.11, *SE* = 0.03, 95% CI = 0.05, 0.16), confirming that the relationship between weight stigma and the tendency to avoid physical activity and sport is through WBIS and FNAE (serial mediation).

## 4. Discussion

Participation in physical activity and sport is important for the physical, social, and mental wellbeing of the general public, yet participation has been declining for several decades. The present exploratory study sought to better understand the psychosocial factors that may help explain why people may not enjoy or seek to avoid participating in physical activity and sport. Previous research in this area had found a relationship between weight stigma and exercise avoidance [16,41]; however, no work has assessed the potential psychosocial factors underpinning the relationship between weight stigma and enjoyment or tendency to avoid physical activity and sport. Accordingly, the present exploratory study addresses this gap by examining the relationships between weight stigma and appearance-related concerns and enjoyment and tendency to avoid physical activity and sport.

This study found that weight stigma, weight stigma internalization and appearance evaluation were all associated with lower enjoyment of, and tendency to avoid, physical activity and sport. Additionally, fear of negative appearance evaluation was associated with the tendency to avoid physical activity and sport, but not with the enjoyment of physical activity and sport after accounting for all other variables in models. Furthermore, the relationship between weight stigma and enjoyment of physical activity and sport was mediated by body image (appearance evaluation) and weight bias internalization. The serial mediation analysis showed that experiences of weight stigma were associated with lower appearance evaluation, which was associated with greater weight bias internalization, which in turn was associated with less enjoyment of physical activity and sport. Similarly, mediation analyses showed that greater weight stigma experience was associated with greater weight bias internalization, which was associated with greater fears of negative appearance-related evaluations, which was related to tendency to avoid physical activity and sport scores.

Consistent with previous research, weight bias internalization had a stronger relationship with lower enjoyment and tendency to avoid physical activity and sport than did experiences of weight stigma [42]. This suggests that weight stigma experiences may drive negative thoughts about one’s own weight (weight stigma internalization), which then underpin lower enjoyment of physical activity and sport and tendency to avoid physical activity and sport. This is an important finding as it suggests that not everyone who encounters weight stigma will develop other negative psychosocial and behavioral sequela, but those who internalize such stigmatizing comments and behaviors may encounter these negative outcomes. However, our findings appear to suggest that if negative verbal comments and teasing about weight are internalized by the individual, then the impact of weight stigma is amplified. This also suggests that there may be a window for intervention following experiences of stigma to reduce its impact. For example, acceptance commitment therapy shows promise in this regard, but more research on the effectiveness of this sort of approach is needed [43]. Research is also needed to identify why some individuals may be more prone to internalizing weight stigma, and who is at greatest risk for internalization and its negative implications for engagement in physical activity and sport [12].

It is also worth noting that those with greater appearance evaluation reported lower enjoyment of physical activity and sport. But again, enjoyment also appears to be related to experiences of being stigmatized about one’s appearance or weight. Similarly, fear of negative appearance-related evaluations from others was related to the tendency to avoid physical activity and sport, but not to enjoyment of physical activity and sport. It may be that individuals enjoy physical activity and sport once they are engaged in the activity, but fear of being negatively judged about their appearance acts as a potential barrier to participating.

These findings reiterate the need for inclusive physical activity and sport policy and weight stigma interventions, particularly those that target the internalization of negative weight-related commentary. Previous research has discussed the importance of policies to reduce weight-based discrimination [44,45], with the aim of making weight stigma less socially acceptable [46]. Our findings indicate the importance of targeting physical activity and sport settings as part of these efforts. More broadly, our findings support calls for public health approaches to develop and support environments that encourage health-promoting behaviors for people across diverse weights and body sizes. Current public health approaches and messaging to increase people’s physical activity levels typically focus on weight status (e.g., obesity, overweight) and associated health consequences of poor diet and exercise, which by association can increase weight stigma [47].

There are limitations to the present research. The cross-sectional design of the study prohibits strong causal inferences regarding the pathways between weight stigma and enjoyment and tendency to avoid physical activity and sport. A longitudinal study would be optimal to test causal relationships between weight stigma, enjoyment, and tendency to avoid physical activity and sport, especially given that the onset of weight stigma experiences can occur very early in life [48]. Related work by others does suggest that the relationships identified in the present study are likely to be present in longitudinal research [49,50]. The age of the sample is relatively homogenous with the majority of the sample consisting of young adults. Body image concerns and related constructs tend to decrease over time and may have less predictive power in older adults [51,52]. Future research should include samples of older individuals to understand if the relationships observed here are present in later life. Weight stigma was also not assessed within the specific context of physical activity and sport, which should be examined in future research. Finally, the present study focused on experiences and feelings about participating in physical activity and sport rather than participation and physical activity behaviors per se. However, it seems reasonable to expect that those reporting not enjoying, and wanting to avoid, physical activity and sport will be less likely to participate in it.

## 5. Conclusions

Notwithstanding the limitations, the study results suggest that enjoyment and the tendency to avoid physical activity and sport are related to experienced and internalized weight stigma, appearance evaluations, and fear of negative appearance evaluations. Further research is required, such as longitudinal studies, which can better examine the causal relationships between weight stigma, weight stigma internalization, appearance evaluations, fear of negative appearance evaluation, and enjoyment and tendency to avoid physical activity and sport. Interventions to understand the causal relationships between these variables would further assist research in the psychological and public health fields in understanding what can be done to reduce physical activity and sport avoidance. Understanding the psychosocial barriers to physical activity and sport is key for improving the population’s engagement and adherence to these activities, and to improving the social, mental, and physical well-being of the population.

## Figures and Tables

**Table 1:** Mean (*SD*) participant ratings for men and women on each of the variables along with *t*-values, significance levels, score ranges, and effect sizes for group differences.

Variables	Men (*N* = 151)	Women (*N* = 427)	Total	Minimum–Maximum	*t* Value	*p* Value	Effect SIZE ^†^ *d’*
Age	20.21 (1.92)	19.70 (2.20)	19.83 (2.14)	17–35	2.54	0.011	0.26
BMI	23.22 (3.20)	22.22 (3.49)	22.48 (3.45)	14.88–38.74	3.10	0.002	0.30
Weight stigma	6.00 (4.29)	6.49 (4.83)	6.36 (4.70)	2.50–23	−1.09	0.276	0.11
WBIS	2.60 (1.17)	3.30 (1.39)	3.12 (1.37)	1–7	−5.39	0.000	0.52
Appearance Evaluation	3.49 (0.76)	3.17 (0.76)	3.25 (0.77)	1–5	4.36	0.000	0.24
FNAE	16.23 (5.70)	18.98 (6.37)	18.26 (6.32)	6–30	−4.70	0.000	0.45
Enjoyment	4.12 (0.66)	3.72 (0.77)	3.82 (0.76)	1.31–5	5.34	0.000	0.55
TAPAS	16.16 (8.66)	20.86 (10.40)	19.63 (10.18)	10–50	−4.92	0.000	0.50

Note: ^†^ Cohen’s *d’*, small effect ≈ 0.2 moderate effect ≈ 0.5, large effect ≈ 0.8+. Weight stigma = Perception of teasing scale, WBIS = Weight bias internalization scale, Appearance evaluation = Self-appearance evaluation, FNAE = Fear of negative appearance evaluation, Enjoyment = The physical activity enjoyment scale, TAPAS = Tendency to avoid physical activity and sport.

**Table 2:** Pearson’s product moment correlations between all variables. Correlations for men (*N* = 151) are displayed above the diagonal, and women (*N* = 427) are displayed below the diagonal.

	1	2	3	4	5	6	7	8
1. Age	-	0.07	−0.03	0.01	0.09	−0.08	0.19 *	−0.14
2. BMI	0.01	-	0.17 *	0.23 **	−0.21 **	−0.04	0.12	0.14
3. Weight stigma	0.03	0.24 ***	-	0.60 ***	−0.45 ***	0.39 ***	−0.28 **	0.46 ***
4. WBIS	0.02	0.35 ***	0.51 ***	-	−0.75 ***	0.54 ***	−0.33 ***	0.58 ***
5. Appearance evaluation	0.07	−0.32 ***	−0.41 ***	−0.71 ***	-	−0.43 ***	0.36 ***	−0.51 ***
6. FNAE	−0.06	0.10 *	0.31 ***	0.62 ***	−0.55 ***	-	−0.18 *	0.35 ***
7. Enjoyment	0.03	−0.07	−0.11 *	−0.27 ***	0.27 ***	−0.19 ***	-	−0.50 ***
8. TAPAS	−0.04	0.26 ***	0.50 ***	0.60 ***	−0.47 ***	0.50 ***	−0.33 ***	-

* p < 0.05, ** *p* < 0.001, *** *p* < 0.0001.

**Table 3:** Regression models reporting unstandardized (*B*) and standardized beta’s (*β)* and standard errors (*SE*) for predictors of the enjoyment of physical activity and sport, and the tendency to avoid physical activity and sport measures.

Enjoyment of Physical Activity and Sport																				
	Model 1					Model 2					Model 3					Model 4				
	*B*	*SE*	*β*	*CI*		*B*	*SE*	*β*	*CI*		*B*	*SE*	*β*	*CI*		*B*	*SE*	*β*	*CI*	
Age	0.02	0.02	0.06	−0.01	0.05	0.02	0.02	0.06	−0.01	0.05	0.02	0.01	0.05	−0.01	0.04	0.02	0.01	0.05	−0.01	0.05
Sex	−0.37	0.07	−0.22 ***	−0.52	−0.23	−0.34	0.07	−0.20 ***	−0.49	−0.20	−0.20	0.07	-0.12 *	−0.34	−0.05	−0.20	0.07	−0.12 *	−0.34	−0.05
BMI	−0.01	0.01	−0.03	−0.03	0.01	0.00	0.01	0.01	−0.02	0.02	0.02	0.01	0.09 *	0.00	0.04	0.02	0.01	0.09 *	0.00	0.04
WS	-					−0.03	0.01	−0.16 ***	−0.04	−0.01	5.13	0.01	0.00	−0.02	0.02	−2.58	0.01	0.00	−0.02	0.02
AE	-					-					0.19	0.06	0.19 **	0.07	0.30	0.19	0.06	0.20 **	0.08	0.31
WBIS	-					-					−0.10	0.04	−0.19 *	−0.17	−0.04	−0.11	0.04	−0.20 *	−0.19	−0.35
FNAE	-					-					-					0.00	0.01	0.02	−0.01	0.02
*R* ^2^	0.05 ***					0.08 ***					0.16 ***					0.16 ***				
**Tendency to Avoid Physical Activity and Sport**																				
Age	−0.35	0.19	−0.07	−0.73	0.03	−0.39	0.17	−0.08 *	−0.72	−0.05	−0.37	0.16	−0.08 *	−0.68	−0.07	−0.33	0.15	−0.07 *	−0.63	−0.03
Sex	5.53	0.96	0.24 ***	3.6	7.41	4.6	0.85	0.20 ***	2.98	6.31	1.98	0.80	0.09 *	0.41	3.55	1.98	0.79	0.09 *	0.43	3.53
BMI	0.71	0.13	0.23 ***	0.47	0.96	0.36	0.12	0.12 *	0.13	0.58	0.05	0.12	0.02	−0.17	0.26	0.13	0.12	0.04	−0.08	0.34
WS	-					1.0	0.80	0.46 ***	0.84	1.15	0.53	0.08	0.25**	0.37	0.70	0.53	0.08	0.25 **	0.37	0.69
AE	-						-				−0.71	0.64	−0.05	−1.96	0.54	−0.33	0.64	−0.03	−1.58	0.92
WBIS	-						-				3.16	0.38	0.42 **	2.41	3.91	2.52	0.41	0.34 **	1.71	3.33
FNAE	-						-				-					0.26	0.07	0.16 **	0.13	0.40
*R* ^2^	0.10 ***						0.30 ***				0.44 ***					0.45 ***				

Note: Sex uses men as the reference group. WS = Weight Stigma, AE = Appearance Evaluation, All VIF’s for all models were below 3.0. * *p* < 0.05, ** *p* < 0.001, *** *p* < 0.0001.

**Table 4:** Regression coefficients and standard errors for the two serial mediation models presented in Figure 1 and Figure 2 and testing mediating relationships with enjoyment and avoidance of sport and physical activity respectively. All models account for age, BMI, and sex.

	Consequent											
		Appearance Evaluation				WBIS				Enjoyment		
**Antecedent**		**Coeff.**	***SE***	***p***		**Coeff.**	***SE***	***p***		**Coeff.**	***SE***	***p***
Weight stigma	*a_1_*	−0.06	0.01	<0.000	*a_2_*	0.07	0.01	<0.000	*c’*	0.00	0.01	0.99
Appearance evaluation		-	-	-	*d_12_*	−1.03	0.06	<0.000	*b_1_*	0.19	0.06	0.00
WBIS		-	-	-		-	-	-	*b_2_*	−0.10	0.04	0.00
		**WBIS**				**FNAE**				**TAPAS**		
**Antecedent**		**Coeff.**	***SE***	***p***		**Coeff.**	***SE***	***p***		**Coeff.**	***SE***	***p***
Weight stigma	*a_1_*	0.13	0.01	<0.000	*a_2_*	0.03	0.05	0.52	*c’*	0.54	0.08	<0.000
WBIS		-	-	-	*d_12_*	2.99	0.19	<0.000	*b_1_*	2.58	0.36	<0.000
FNAE		-	-	-		-	-	-	*b_2_*	0.27	0.07	<0.000

Note: Weight stigma = Perception of teasing scale, WBIS = Weight bias internalization scale, Appearance evaluation = Self-appearance evaluation, FNAE = Fear of negative appearance evaluation, Enjoyment = The physical activity enjoyment scale, TAPAS = Tendency to avoid physical activity and sport.

## Data Availability

Data from the present study can be requested from the corresponding author Kerry O’Brien (kerrykez@gmail.com).
